# Comparison of the Low-Velocity Impact Responses and Compressive Residual Strengths of GLARE and a 3DFML

**DOI:** 10.3390/polym15071723

**Published:** 2023-03-30

**Authors:** Ke Wang, Farid Taheri

**Affiliations:** Advanced Composites and Mechanics Laboratory, Department of Mechanical Engineering, Dalhousie University, Halifax, NS B3H 4R2, Canada

**Keywords:** 3DFML, GLARE, compression after impact, low-velocity impact, finite element analysis

## Abstract

The impact performance and compression after impact characteristics of 2D and 3D fiber metal laminates (FMLs) are investigated both experimentally and numerically. Commercial-grade GLARE3A-3/2-0.3, and a recently developed FML, which incorporates a unique 3D glass fabric, are used in the study. Both FMLs have similar areal densities. The specimens are subjected to impact loading at three energy levels—low, intermediate, and high. The test results indicate that GLARE is slightly more resilient under impact compared to the 3DFML. However, since GLARE is much thinner than the 3DFML, the two-material systems exhibit very different failure modes. GLARE and 3DFML lost up to 62.6% and 41.5% of their original compressive load-bearing capacity, respectively. Robust and accurate finite element models are developed that can predict the damage evolution and failure modes of both FMLs. Knowing the level of reduction in the residual load-bearing capacity of a material resulting from an impact is of practical importance when assessing the service life of materials. However, further exploration would be required to determine how the information obtained through testing relatively small-sized specimens in a laboratory environment can be extrapolated to larger real-life structural components.

## 1. Introduction

Fiber metal laminate (FML) is a hybrid laminated material system consisting of layers of fiber-reinforced plastics (FRPs) and thin sheets of metal. FML was developed with the aim of taking advantage of the positive attributes of the metallic and FRP constituents. Since their introduction, various types of FMLs have been developed (e.g., ARALL, GLARE and CARLL). FMLs offer outstanding static properties, impact resistance and fatigue resistance, and they have primarily been used in the aerospace industry. For example, glass-laminated aluminum-reinforced epoxy (GLARE), possibly the most widely used FML type, has been successfully incorporated in the fuselage of the Airbus A380 airliner [1]. A few years ago, a novel class of FML was developed by our research group, referred to as 3D fiber metal laminates (3DFMLs). Since its inception, the material system has undergone a systematic series of experimental and numerical investigations to establish its behavior under quasi-static (including buckling response) [2], low-velocity out-of-plane impact [3,4], in-plane impact [5,6], and joining techniques [7,8,9]. 3DFMLs have excellent stiffness and strength-to-weight ratios due to their unique internal geometry and configuration, making them a strong candidate for applications in various industries.

Aerospace and transport vehicles’ structural components are susceptible to low-velocity impact (LVI) loadings during their fabrication, assembly, operation and maintenance [10]; examples include dropped tools, collisions with cargo or service vehicles, impacts of runway debris and ice, etc. [11]. The low-velocity impact is especially detrimental to composites because not only could it cause significant damage to the material that would invariably compromise the structure’s integrity, but it could also cause significant invisible internal damage that is undetectable during routine visual inspections [12,13]. In the industry, this type of damage is referred to as “Barely Visible Impact Damage” (BVID). Therefore, a material’s impact resistance and tolerance to impact damage are critical for their incorporation into industrial applications, especially in the aerospace industry.

The residual load-carrying capacity of a composite after impact loading is usually evaluated using the standardized testing method, referred to as the compression after impact (CAI) tests, such as those outlined in ASTM D7137 and ISO 18352 [14,15]. These tests were originally developed to assess the residual strength of carbon-based fiber-reinforced plastics (CFRP). The ASTM CAI is generally carried out in conjunction with ASTM D7136 (drop-weight low-velocity impact test) and ASTM D6641 (pure compression test) [16,17].

It should be noted that the majority of the literature on this topic has focused on the CAI performance of composite materials (mainly CFRP), and only a few works have investigated the CAI performance of FMLs. For instance, Wang et al. [18] investigated the CAI performance of quasi-isotropic composite laminates and concluded that internal delamination could be more critical than near-surface delamination. Vieille et al. [19] conducted a comparative study to examine the influence of matrix toughness and ductility on the CAI performance of FRPs. This was achieved by comparing the response of epoxy-based FRPs to PPS- and PEEK-based FRPs. Their results revealed that the matrix’s toughness and ductility had minor effects on FRP’s CAI performance, even though the different toughness and ductility of the matrix changed the damage growth path. Ghelli and Minak [20] investigated the CAI performance of thin carbon/epoxy laminates. The specimens were restrained during the impact event using a circular fixture and the standard rectangular impact fixture specified in ASTM D7136. The results indicated that the circular fixture opening eliminated the edge effect caused by the shorter side of the rectangular fixture opening. They also stated that the testing standard should not be used to establish the material’s post-impact compressive strength; instead, the test method would be suitable to characterize the post-impact compressive response of laminates, capturing the global or local instability responses of the material. Rivallant et al. [21] incorporated cohesive elements in their finite element model to predict the crack propagation developed in their CFRP specimens during CAI and their failure mode. Yang et al. [22] investigated the CAI performance of CFRP and claimed that the laminate’s strength decreased by approximately 34–53% after it had been subjected to impact energies in the range of 20–40J. Dhaliwal and Newaz [11] studied the CAI response of carbon fiber-reinforced aluminum laminates (CARALL). They successfully used cohesive tiebreak contact to predict the delamination and failure mode of CARALL. They also observed that the residual strength of their CARALL decreased by 28–41% after being subjected to impact energy of 31 J.

All in all, limited data are available regarding the CAI response of FMLs (especially for GLARE) and are virtually non-existent for 3DFMLs. As a potential candidate for applications in aerospace and other transport industries, the CAI characteristics of 3DFMLs would have to be investigated and compared to the current commercial-grade FMLs (e.g., GLARE). Therefore, in this study, LVI and CAI responses of 3DFML and GLARE are experimentally investigated and compared. To complement the investigation, models based on the finite element method (FEM) are developed to simulate the two experimental procedures. The integrity of the models is validated against the experimental results. These models could be effectively utilized to examine the influence of various parameters that influence the response of such complex hybrid material configurations and optimize their performance.

It is believed that the in-depth experimental and numerical investigations presented in this paper serve as a valuable attempt to fill this knowledge gap in the literature. Moreover, knowledge about the reduction in the residual load capacity of such materials after an impact would be beneficial to practicing engineers. It will also be demonstrated that micro-CT scanning is a highly effective technique for gaining insight into the performance of hybrid composites with complex configurations.

## 2. Materials

**Figure 1:**
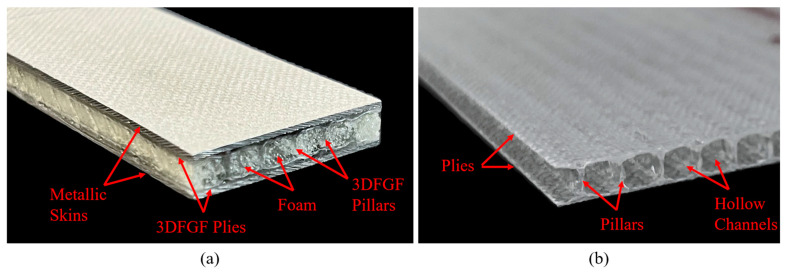
(**a**) 3DFML and (**b**) 3DFGF layouts.

Figure 1a shows the configuration of 3DFML. The unique 3D fiberglass/epoxy composite (3DFRP) constituent of the 3DFML is illustrated in Figure 1b. The 3D fiberglass fabric (3DFGF) constituent consists of upper and lower biaxial glass fabrics interlaced by a series of glass fibers referred to as pillars. In this study, the hollow channels between the biaxial fabrics are filled with polyurethane foam to enhance the through-thickness performance of the core structure. 3DFMLs are made by laminating metallic layers to the 3D composite core, as shown in Figure 1a.

The first step of producing the 3DFML panels involved impregnating a $240\times 190\;{mm}^{2}$ piece of 3DFGF (supplied by Beihai Fiberglass, Jiujiang City, China) using the West System room-cured 105/206 epoxy resin/hardener system (Bay City, MI, USA). The fabrication processes of 3D spacer composite are as follows.

First, a layer of epoxy resin was applied evenly onto a peel-ply that hosted the 3DFGF. Then, epoxy resin was brushed onto the 3DFGF against the pillar yarn’s direction; this brushing process was repeated after flipping the fabric over. Finally, a layer of peel-ply was placed on top of the fabric, and the whole assembly was lightly brushed against the pillar yarn direction, before being cured at room temperature for 24 h.

In the second step, a two-part liquid polyurethane foam with a density of $256\;kg/m^{3}$ (US Composites, Palm Beach, FL, USA) was mixed and injected into the cavities of the 3D composite using the vacuum-assisted resin transfer molding technique. Lastly, the mating surfaces of two 0.5 mm thick AZ31B-H24 magnesium alloy layers (acquired from MetalMart, Commerce, CA, USA) were degreased with acetone, rinsed, and dried with compressed air. The surfaces were then grit blasted using aluminum oxide and subsequently rinsed, dried, and once again wiped with acetone. The sheets were then bonded to the 3D composite using the same resin and left to cure for 36 h under a 1 bar vacuum. The nominal thickness of 3DFML was about 5.2 mm.

Since most applications that utilize FMLs are weight-sensitive, a lightweight grade of GLARE was selected to match the areal density of the 3DFML. Specifically, a commercial-grade GLARE3A-3/2-0.3 (with the layup of ${\left[Al/0{}^{\circ}/90{}^{\circ}/\overset{-}{Al}\right]}_{s}$) was used in this study. The metallic layers of this FML were 0.3 mm thick 2024-T3 aluminum sheets, and the FRP layers were 0.125 mm thick unidirectional S-glass/Epoxy prepregs, yielding an overall approximate thickness of 1.4 mm.

## 3. Experiments

Appropriate size specimens were extracted from the panels using a diamond-edged rotary saw. Each specimen was first subjected to a through-thickness low-velocity impact and was subsequently tested under quasi-static compression, following ASTM D7136 and ASTM D7137 guidelines, respectively.

### 3.1. Impact Testing Setup

**Figure 2:**
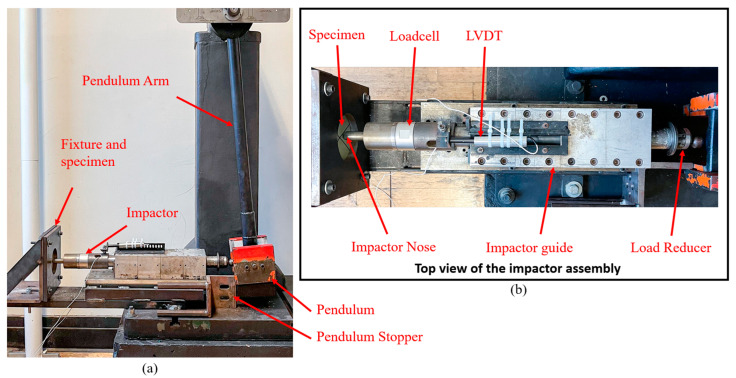
Low-velocity impact test setup. (**a**) Overview of the impact equipment; (**b**) a close-up view of the impactor assembly.

A Charpy impact testing equipment was modified to generate the desired low-velocity impact (see Figure 2a). It consists of a pendulum, an impactor, a bearing-fitted impactor guide, a pendulum stopper, and a fixture that holds specimens. A closer view of the impactor and impactor guide assembly is shown in Figure 2b. The impactor moves in the horizontal direction with the guidance of the impactor guide. Linear bearings were used between the impactor and the impactor guide to minimize the energy loss. A Dytran 1060 dynamic load cell was placed near the impactor’s nose to measure the force response. The impactor has a round nose with a radius of 8 mm. A dynamic LVDT was used to measure the real-time displacement of the impactor. The outer shell of the LVDT was fixed on the impactor guide, while the inner cylinder was attached to the impactor. Two high-speed cameras, a Photron Fastcam PCI and a Kron Chronos 1.4, were used to record the impact events.

During an impact test, the pendulum sways down from an elevated position and hits the impactor, transferring energy to the impactor and propelling the impactor toward the specimen. The impactor subsequently hits the specimen, and the effective data are captured at this stage. Due to the large inertia of the pendulum, the pendulum was observed to keep travelling forward after its first impact with the impactor. After the impactor hits the specimen, the impactor either travels slower than the pendulum (in cases when perforation occurs) or rebounds back (for non-perforation cases), giving the pendulum a chance to hit the impactor a second time. This is critically harmful to this experiment because additional energy would be transferred to the specimen, adversely affecting the consistency of the subsequent phase residual strength evaluation. The second hit between the pendulum and the impactor usually occurs so quickly after the initial impact that the naked eye cannot observe it; this event could only be detected by the joint observation of the high-speed camera footage and the force response data. The unwanted second impact was effectively eliminated after implementing a suitable pendulum stopper. The pendulum stopper is a steel piece that limits the pendulum’s travel beyond hitting the impactor. As a result, the pendulum stops swaying forward after transferring energy to the impactor, and the impactor hits the specimen only once and bounces back to its initial position.

Additionally, a load reducer made of rubber and a steel protector were fitted to the back of the impactor. Initially, it was observed that the peak load occurred when the pendulum hit the impactor, rather than when the impactor hit the specimen (due to the steel-to-steel impact). This phenomenon is problematic for two reasons. Firstly, the maximum impact load that can be accurately measured is limited by the working limit of the load cell. Moreover, in an event when an impact load exerts an excessive peak, its load would compromise the impact energy capacity of the testing equipment. Secondly, it was observed that the metal-to-metal impact excited the impactor and caused “rougher” load signals. Adding the load reducer significantly decreased the peak load and made the load curve smoother. An advantage of using this pendulum-type setup over the commonly used drop-weight impact setup is that the energy is singular to height, and no additional treatment is required for correcting the gravitational force. Therefore, the refined test setup is believed to be equivalent to (if not more effective than) the drop-weight impact testing setup, as per ASTM D7136.

Furthermore, an impact fixture with a circular opening was adopted instead of the standard fixture with a rectangular opening. A circular opening ensures uniformly distanced boundary conditions with respect to the point of impact, thereby eliminating the edge effect; in other words, the support would potentially enhance the consistency in capturing the anisotropic response of the composite panels. The specimens were sandwiched between the front and back fixture plates, which were bolted together at all four corners. The circular opening had a diameter of 85 mm.

In this study, the considered energy levels were 10 J, 20 J, and 30 J for 3DFML and 10 J, 25 J, and 40 J for GLARE. These energies were established by conducting a series of initial numerical simulations similar to those which will be presented later. The three impact energy levels of each material system are referred to as low-, intermediate-, and high-energy levels hereafter in this paper. For each material category, the low-energy level corresponds to minimal damage (commonly referred to as barely visible damage), the intermediate-energy level generates moderate damage (damage visible on the front or back surface of the specimen), and the high-energy level generates severe damage (complete or partial penetration).

The impact energies can be determined using Equation (1) for the given experimental setup.

(1) $$E=\frac{1}{2}mv^{2}$$

where m is the impactor’s mass (5.822 kg), and v is the impactor’s velocity. To ensure the accuracy of the recorded energy values, the impactor’s velocity was measured by processing the LVDT data, the portion captured just before the impactor hit the specimen. The magnitude of velocity used in calculating the energy was established using Equation (2).

(2) $$v=\frac{z_{1}-z_{2}}{t_{1}-t_{2}}$$

where $z_{1}$ and $z_{2}$ are the displacement values selected at the onset of impact and at a point selected on the linear portion of the graph of displacement vs. time (i.e., $t_{1}$ and $t_{2}$), respectively. The impact equipment was calibrated using Equation (3) to relate the pendulum arm’s swing angle and the impact energy before conducting the actual experiments.

(3) $$E=\frac{1}{2}mv^{2}=c_{1}\left(1-\cos\theta \right)+c_{2}$$

In the above equation, θ is the pendulum arm’s swing angle measured from the vertical axis, and $c_{1}$ and $c_{2}$ are the constants determined from the calibration (i.e., determined by a linear least-square fit to plot the measured kinetic energy of the impactor versus (1−cos⁡θ)). The calibrated pendulum equipment can accurately carry out LVI to a specimen with any specified energy up to 45 J.

### 3.2. Micro-CT Scanning

All specimen groups were assessed for damage after impact before each specimen was subjected to the compression test. There are many non-destructive inspection methods for assessing damage in composite materials, including visual inspection (VI) and other techniques, such as ultrasonic, thermography, radiography, electromagnetic, acoustic emission, and stereography, which require specialized equipment [23]. This study used a BRUKER Skyscan 1276 micro computerized tomography (micro-CT) scanner to ascertain the internal damage and its severity. A micro-CT scan uses X-rays to scan 360° around an object, producing images of detailed internal structures. The resulting images can present the cross-section views of the specimen (without the need for sectioning the specimen) and can be taken in any arbitrary orientation and position. Micro-CT has been proven to be effective in observing the internal damage of composite panels [24,25]. The set emission parameters for the scans were 55 kV and 200 μA while using a 0.25 mm thick aluminum filter, and the voxel size was 42 μm; these parameters were established as optimal for GLARE and 3DFML through several trials.

### 3.3. CAI Test

**Figure 3:**
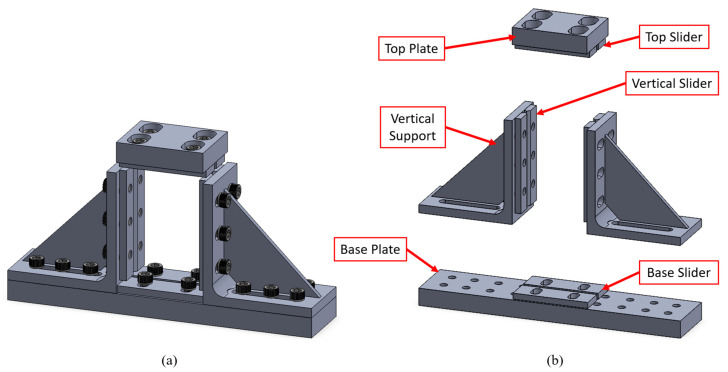
CAI fixture (**a**) assembled; (**b**) exploded view.

After the impacted specimens were scanned, they were subjected to quasi-static compression tests to establish their residual performance. According to ASTM D7137, the CAI test requires a unique compression fixture, as shown in Figure 3. This compression fixture consists of 10 parts. The impacted specimen with a dimension of 100 mm×150 mm is inserted into the compression fixture with the longer edge being vertical. The top plate is rested on top of the specimen, and sliders are subsequently adjusted to accommodate the impacted specimen as per the standard. Note that the top plate is not attached to the assembly and can move relative to the assembly to compress the specimen. Moreover, the vertical sliders were machined to produce single-edge (lateral) support; in other words, they do not restrain the specimen’s vertical edges against rotation or sliding. In contrast, two base sliders and two top sliders are bolted onto the base plate and the top plate, respectively, and are in contact with the specimen to provide out-of-plane restraining support and reduce the stress concentration at the top and bottom edges of the specimen.

The ultimate strength of the damaged specimen can be determined using Equation (4) [14].

(4) $$F^{CAI}=\frac{P_{max}}{A}$$

where $P_{max}$ is the maximum compression force applied to the specimen by the test machine, and A is the cross-section area of the specimen. During the compression test, a laser extensometer is used to measure the strain at an undamaged area of the specimens. The effective modulus is established using the following equation.

(5) $$E^{CAI}=\frac{P_{\epsilon_{2}}-P_{\epsilon_{1}}}{A\times \left(\varepsilon_{2}-\varepsilon_{1}\right)}$$

where $\varepsilon_{1}$ and $\varepsilon_{2}$ are recorded strains at two stages of loading, $P_{1}$ and $P_{2}$, respectively.

In total, 13 3DFML and 9 GLARE specimens with dimensions of 100 mm×150 mm were prepared for CAI experiments.

### 3.4. Compression Response of the Virgin (Undamaged) Specimens

**Figure 4:**
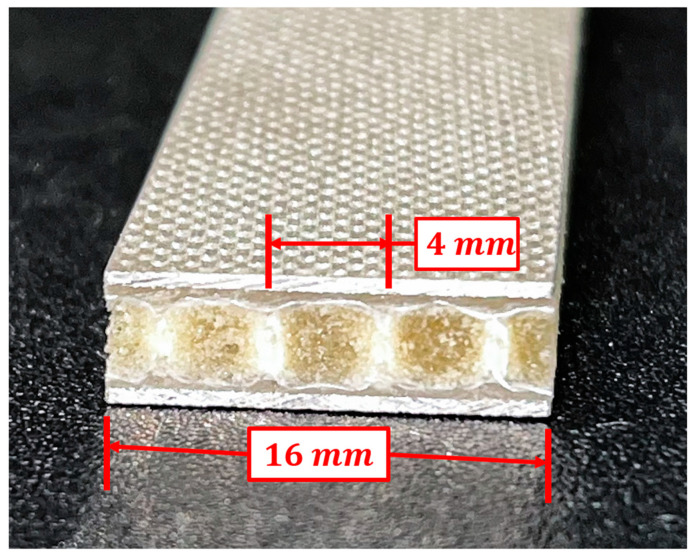
Customized compression test specimen width for 3DFML.

The compressive response of the virgin specimens was also evaluated following ASTM D6641. All specimens were 140 mm in length. GLARE specimens were 12.7 mm in width, as per the standard, while 3DFML specimens were 16 mm in width. The different width of 3DFML specimens is because the specimen’s width should be a product of 4 mm. Furthermore, the width of 16 mm was chosen instead of 12 mm because the larger width can reduce the potential influence of the cutting-induced tolerances. Figure 4 illustrates the cross-section of a 3DFML specimen prepared for the virgin compression test.

The minimum thickness of each material category was established based on the layup. In the standard, the thickness of specimens must satisfy Equation (6) to preclude the Euler buckling of the specimen.

(6) $$h\geq \frac{l_{g}}{0.9069\sqrt{\left(1-\frac{1.2F^{cu}}{G_{xz}}\right)\left(\frac{E^{f}}{F^{cu}}\right)}}$$

where $l_{g}$ is the gage length, $F^{cu}$ is the estimated ultimate compressive strength, $G_{xz}$ is the through-thickness shear modulus and $E^{f}$ is the estimated flexural modulus. The mentioned thicknesses of GLARE and 3DFML satisfy the above equation, using the 13 mm gauge length ($l_{g}$) recommended by the standard.

The special fixture used to support the specimen in the compression test has two halves, gripping the top and bottom sections of the specimen. The two halves are aligned concentrically by two rails (see ASTM D6641). The upper half slides on the rails effortlessly via bearings, thereby subjecting the specimen to a concentric compressive load with buckling prevented by the established gauge length. A laser extensometer was used to record the resulting strain in the gauge length. Five specimens of each system were prepared for the compression tests.

## 4. Numerical Simulations

The LS-DYNA commercial software package was used to conduct the numerical simulations. Specifically, LS-PrePost V4.9.10 and solver R13.1.1 were used in this study. In addition, the Message Passing Parallel (MPP) solver was used for all the analyses to optimize the running time. The analyses were run using 150 cores and 4 gigabits of memory for each core on the Cedar cluster provided by Digital Research Alliance of Canada.

### 4.1. FE Models

**Figure 5:**
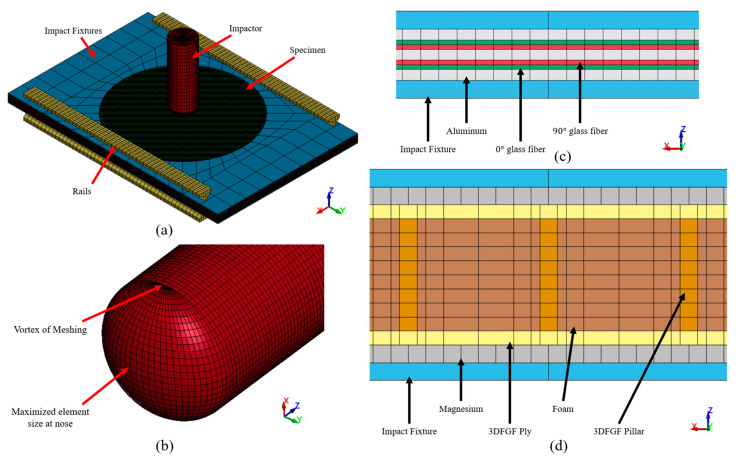
Numerical model: (**a**) overall model layout; (**b**) mesh of the impactor nose; cross-section mesh of (**c**) GLARE and (**d**) 3DFML.

The FE models of the FMLs are shown in Figure 5. The specimen was sandwiched between two impact fixtures, and the impactor was initially placed at a distance of 1 mm from the specimen. Impact fixtures and the impactor were modelled using solid elements (ELFORM = 1) and “rigid” material. To restrain a part constructed using *MAT_RIGID in LS-DYNA, the constraints must be applied in the material card instead of the commonly used BOUNDARY_SPC option. The impact fixtures were fully restrained in the *x*, *y*, and *z* directions, and the impactor was restrained in the *x* and *y* directions. The impactor’s material density was selected to match the actual impactor’s mass. The mesh of the impactor’s nose is shown in Figure 5b. The element sizes are strategically selected to control the critical timestep, thereby enhancing the contact’s numerical stability [26]. The vertical sliders in the CAI tests are simplified as rails in the model.

Figure 5c presents the through-thickness configuration of the mesh modelling the GLARE specimens. It consists of seven layers of thick shell elements (ELFORM = 1), while each thick shell layer corresponded to a specific material layer. Similarly, in the 3DFML model, as shown in Figure 5d, the magnesium, 3DFGF plies, and 3DFGF pillars are modelled using the thick shell element, while the foam was modelled using the solid element (ELFORM = 1). Note that the mesh of the foam becomes progressively refined as it approaches the pillars. It was observed that the out-of-plane compression load on 3DFGFs induced the instability of the pillars while being restrained by the surrounding foam. The finer mesh near the pillars allows for the smoother evolution of instability in the pillars, leading to smoother deletion/erosion of the highly strained elements. To ensure the accuracy of the results and efficient CPU consumption, the specimen’s mesh was kept essentially uniform in the analysis, except at the top and bottom regions, which had coarser meshes due to the relatively lower stress state. In addition, reduced integration elements were used for both thick shell and solid elements to improve the dynamic stability and reduce the computational cost.

### 4.2. Material Models

**Figure 6:**
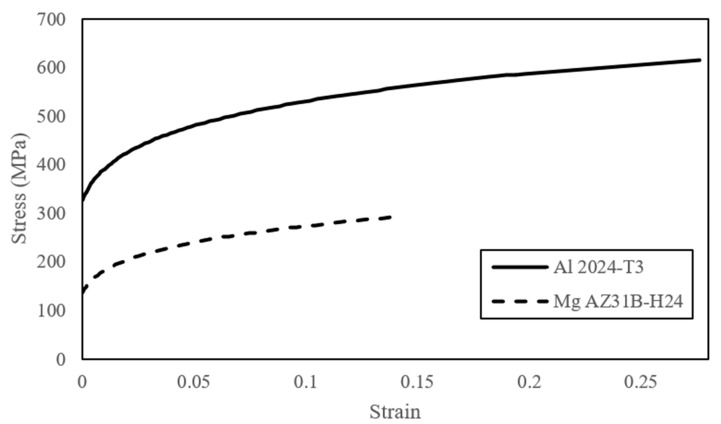
Stress–strain curves representation of the metals’ plasticity.

The metal and FRP constituents of both FMLs were modeled using MAT_24 and MAT_54 of LS-DYNA, respectively. MAT_24 uses a piecewise linear function to define the elastic-plastic behavior of metals and uses a plastic strain-based failure criterion to facilitate element erosion (deletion). The material parameters used in the models are given in Table 1, and the curves that represent the piecewise linear plasticity of the aluminum and magnesium are illustrated in Figure 6.

MAT_54 models the response of an arbitrary orthotropic material effectively in conjunction with the Chang-Chang failure criterion. Notice that the element erosion in MAT_54 is not purely failure-based; instead, element erosion is declared when one of the following criteria is met:

— When the DFAILT parameter is set to zero, the element is deemed to have failed when the tension failure mode is met based on the Chang-Chang stress-based criteria.
— When DFAILT is set to a non-zero value, the element is deemed to have failed if the strain in any direction becomes greater than the defined DFAILT, DFAILC, DFAILM, and DFAILS limits.
— When EFS is set to a positive value, the element is deemed to have failed if the effective strain in the element becomes larger than the set effective failure strain (EFS). EFS is calculated using the ultimate normal and shearing strains.
— When the timestep size criteria for element deletion (TFAIL) is set to a positive value (0 < TFAIL ≤ 1), the element is deemed to have failed when the element timestep is smaller than TFAIL. Conversely, if TFAIL is set greater than 1, the element is deleted when the ratio of the original timestep over the current timestep becomes less than TFAIL.

To correctly model the failure of an FRP part in LS-DYNA, it is essential to define the failure strains of the material. Furthermore, when the second criterion is met, the first criterion (Chang-Chang criterion) would still affect the element’s strength. In such cases, the Chang-Chang criterion parameters define the maximum strengths of the element. In other words, the element would not be eroded (deleted) when the criterion is satisfied; instead, the stress value will hold the element steady at that prescribed maximum strength until the element erodes based on the defined failure strains. For further insight into the definitions of the specific terms, the user is referred to LS-DYNA’s theory manual [27]. The mechanical properties of the FRP constituents are outlined in Table 2.

Lastly, the elastic model, MAT_1, was used for the foam, and an effective strain-based erosion card was adopted to enable the erosion/deletion of the foam elements. The foam has a density of $256\;kg/m^{3}$, an elastic modulus of 120 MPa, Poisson’s ratio of 0.1, and a failure strain of 0.23.

### 4.3. Contact Modelling

The specimen was tied to the two rigid impact specimen-restraining fixtures using TIED_SURFACE_TO_SURFACE_OFFSET, where the “_OFFSET” keyword switches the default constrain-based contact to a penalty-based contact to make tied contact compatible with the rigid material model. When using this option, it is essential to ensure that the slave segment is within the orthogonal projection of the master segment.

In CAI testing, the resulting damaged region of the specimens (the delaminated region) is used to establish the residual strength of the tested specimen. The cohesive zone modelling approach (CZM) is incorporated in this study to model the interfacial delamination growth. The customary method to incorporate CZM in LS-DYNA is to use CZM elements at the region of interest. CZM can also be incorporated by modelling the interface using the surface-to-surface tiebreak contact algorithm. This method enables visualization of the delaminated region during post-processing. To do so, a BINARY_INTFOR card must be included in the input deck, and “*s* = *intfor*” must be included in the command line when executing the analysis. These two steps will produce a series of binary output files named “intfor”. Subsequently, these “intfor” files can be viewed in LS-PREPOST, and the delaminated region can be discerned by fringing the nodal contact gap.

**Figure 7:**
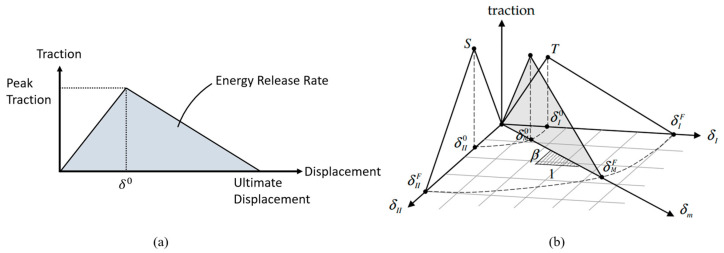
Bilinear CZM models: (**a**) the single-mode model; (**b**) the mixed-mode model [27].

In LS-DYNA, the CZM model MAT_138 (a bilinear mixed-mode relative displacement CZM model) has been adapted into surface-to-surface tiebreak contact as OPTION 9 [28]. Compared to the conventional cohesive element method, this method makes the modelling simpler to operate. This study modelled bonding between the FRP and metallic layers using this method. This modelling approach relates the bonding forces to the relative displacements and considers normal and tangential directions corresponding to Modes I and II fractures, respectively. Figure 7a depicts the bilinear cohesive model in a single fracture mode. In this model, the traction increases linearly with respect to the relative displacement between the two bonded parts. The initial slopes for Mode I and Mode II have the unit of stress/length and are defined as CN and CN×CT2CN, respectively, where CT2CN is the ratio of the stiffness of Mode I to that of Mode II. Once the displacement reaches a critical value ($\delta^{0})$, the traction decreases linearly, simulating the crack growth and the resulting reduction in stiffness. In the tiebreak contact card, the peak tractions for Modes I and II are defined using the normal and shear failure stresses, NFLS and SFLS, respectively; therefore, the critical displacements, $\delta_{I}^{0}$ and $\delta_{II}^{0}$, corresponding to Mode I and Mode II, respectively, can be determined using $\delta_{I}^{0}=NFLS/CN$ and $\delta_{II}^{0}=SFLS/CN/CT2CN$, respectively. Finally, when the traction reduces to zero at the ultimate displacement, it would represent the complete debonding of the interface. The enclosed areas in the bilinear models represent the energy release rates in the corresponding modes and are defined as ERATEN and ERATES, respectively, in LS-DYNA. They represent the areas under the traction/separation curves in Mode I and II, respectively; thus, the ultimate displacements would be ERATEN×2/NFLS and ERATES×2/SFLS.

Understandably, the most realistic debonding would be that caused by the combination of both modes. The mixed-mode bilinear model would consist of a proportion of each mode; this is graphically illustrated in Figure 7b, where β represents the proportionality. The mixed-mode critical displacement, $\delta_{M}^{0}$, and the ultimate displacement, $\delta_{M}^{F}$, are defined by Equations (7) and (8), respectively.

(7) $$\delta_{M}^{0}=\delta_{I}^{0}\delta_{II}^{0}\sqrt{\frac{1+\beta^{2}}{{{\left(\beta \delta_{I}^{0}\right)}^{2}+(\delta_{II}^{0})}^{2}}}$$

(8) $$\delta_{M}^{F}=\frac{2(1+\beta^{2})}{\delta_{M}^{0}}{\left[{\left(\frac{CN}{ERATEN}\right)}^{PARAM}+{\left(\frac{CN\times CT2CN\times \beta^{2}}{ERATES}\right)}^{PARAM}\right]}^{-\frac{1}{PARAM}}$$

The CZM tiebreak contacts were applied to the interfaces between metallic and FRP layers because these interfaces are prone to delamination based on the literature and our experience. The parameters used in the model are summarized in Table 3.

The interfaces between FRP layers and 3DFGF and foam were merged because based on our experience, delamination is less likely to occur in these interfaces. Next, eroding contact was defined between the impactor and the specimen. Eroding contacts were also defined within the specimen between different layers to model the internal interactions more realistically. Lastly, an initial velocity in the negative *z*-direction was applied to the impactor using the INITIAL_VELOCITY_GENERATION card, reflecting the velocity observed in the experiment.

It should be noted that the solution of such a complex system involves an intensive CPU time, which is primarily due to the small contact timestep necessary to ensure solution convergence. To stabilize contacts with a practical global timestep, the SOFT options 1 and 2 were used for cohesive tiebreak contacts and all other contacts, respectively.

### 4.4. Compression Analysis

The dynamic impact analysis was explicitly solved. The quasi-static compression test is a rather lengthy process in real-life; thus, simulating it numerically using the actual loading rate would consume an unfeasibly large CPU. As a result, it would be efficient to use the implicit solver for this phase of the analysis. As mentioned in [11], an effective approach for conducting a seamless static analysis after an explicit dynamic analysis is termed “spring-back analysis” in LS-DYNA. This approach requires the activation of the INTERFACE_SPRINGBACK keyword in the explicit analysis stage. This keyword generates a file called “dynain” upon completion of the explicit (impact) analysis, which contains the final deformation of the elements under the impact loading. Then, an implicit compression analysis input deck should be prepared using the same node numbering as the explicitly analyzed model. The “dynain” file could then be imported into the compression model using the INCLUDE keyword. This enables the initialization of element stresses carried over from the impact analysis at the beginning of the compression analysis.

The described method, however, could not be used because the scheme would not be able to initialize the contact forces, given the fact that bonding interfaces were modelled using “cohesive tiebreak contact” in the current study. Consequently, the static analysis was explicitly continued after the impact simulation to model the compressive loading phase.

At a specific time during an impact simulation, the impact force reduces to zero, indicating the end of the event. This time was used to define the death time for contacts between the impactor and the specimen and between the specimen and the impact fixtures. Boundary conditions associated with the CAI test were then applied to the specimen. First, the regions near the specimen’s top and bottom were all restrained in the *z*-direction. Next, the knife edges of vertical sliders were modeled as rails and were restrained 0.5 mm away from the specimen to replicate the experimental setup (see Figure 5a). Furthermore, the nodes that corresponded to the region of the specimen in contact with the base of the fixture were also restrained in the *y* direction. Then, a prescribed motion was applied to the nodes forming the top edge of the specimen to simulate the compression loading. Lastly, the nodes at the bottom left corner of the specimen were restrained in the *x* direction for the rigid body stability of the model. Ideally, the compressive load (simulated as applied downward displacement here) should be applied using the same rate as in the experimental tests; however, since this phase of the analysis was also solved explicitly (which requires a very small timestep for maintaining solution equilibrium), the adaptation of the actual loading rate in this phase would be extremely CPU intensive, thus impractical. Therefore, the downward (compressive) motion (loading) was applied relatively quickly and as smoothly as possible to make the compression loading phase very short, while minimizing the dynamic inertia effect. Although modelling the applied load based on the actual loading rate would be more accurate, the adopted method has been proven to be viable and effective by other researchers [11]. Note that the same mesh used to model the specimens in the impact analysis phase were used to conduct the CAI, through the boundary conditions, including the contacts between the impactor and specimen and between the impact fixtures and specimens, which were different.

Compression tests conducted on virgin specimens were modelled by reducing the full model to a smaller size to reflect the ASTM D6641 specimen dimensions. Subsequently, appropriate boundary conditions were applied to the model in conformance with the actual specimen gauge length.

### 4.5. Numerical Analysis Control Parameters

Elements can undergo significant deformation during an impact simulation. When the characteristic length of some of the elements decreases during the analysis, the element’s timestep decreases accordingly and causes a decrease in the global timestep. In some cases, the severe deformation of an element can yield a negative volume numerically, attributing a negative timestep to the element and the global system, thus disrupting the progress of the simulation. In simple terms, the severely distorted elements could either dramatically slow down the simulation or cause premature termination of the simulation. To overcome this issue, the ERODE key was set to 1, and the DTMIN was set to 0.01. As a result, the solver would erode the severely distorted elements that require a solution timestep smaller than 1% of the initial timestep used for solving the global model, facilitating the successful completion of the analysis. The timestep scale factor, TSSFAC in the CONTROL_TIMESTEP card, was set at 0.9 because the models were deemed stable. Furthermore, the Flanagan–Belytschko hourglass control (IHQ = 4) algorithm with the hourglass coefficient of 0.1 was incorporated in the model to tame the potential hourglassing phenomenon.

The computation time for these analyses ranged between 5 and 13 h. The analyses that involved energy levels close to the perforation limit of the materials had longer running times, likely because the elements were more severely deformed, resulting in smaller global timesteps. It should be noted that the above time range can only be achieved using a supercomputer facility similar to that used in this study. Based on our experience, the same analyses are estimated to take much longer (on the order of several hundred hours) when conducted on a workstation with an AMD Ryzen-7 CPU, consisting of 8 cores and 3600 Mhz.

## 5. Results and Discussion

### 5.1. Results and Discussion

#### 5.1.1. Experimental Results

**Figure 8:**
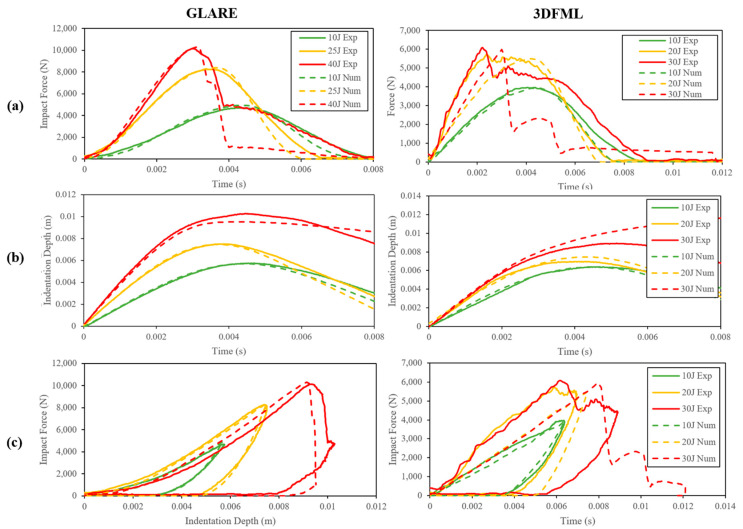
The low-velocity impact test results: plots of (**a**) impact force vs. time; (**b**) indentation depth vs. time; (**c**) impact force vs. displacement.

**Figure 9:**
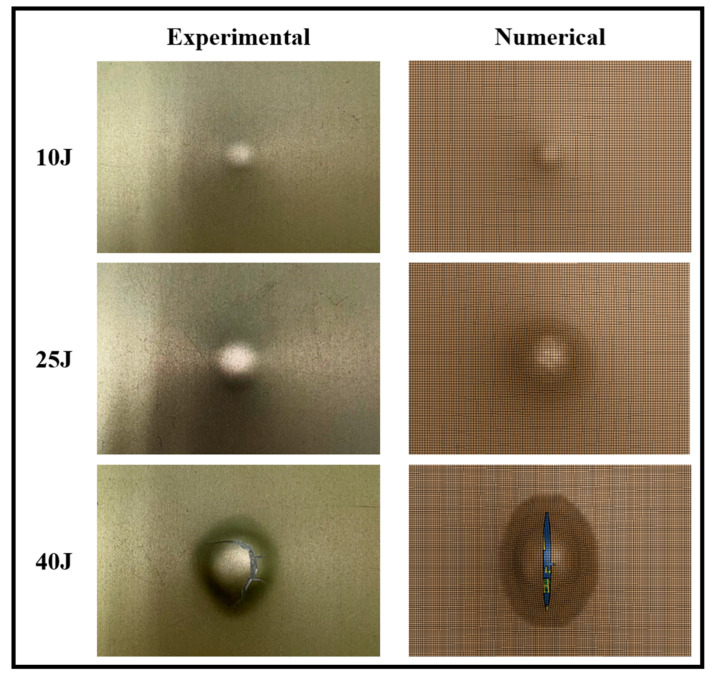
Comparison of the experimental and numerically captured failure modes of the non-impacted surface of GLARE specimens subjected to different low-velocity impact energies.

**Figure 10:**
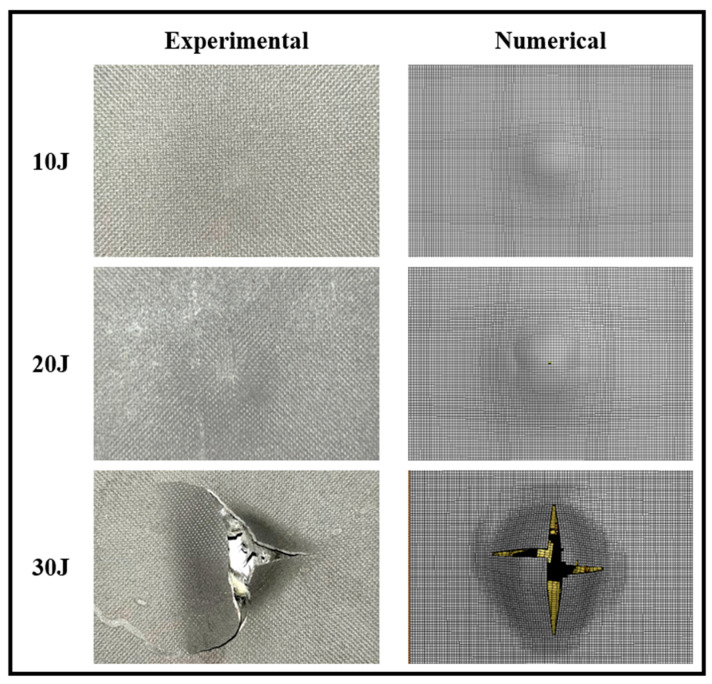
Comparison of the experimental and numerically captured failure modes of the non-impacted surface of 3DFML specimens subjected to different low-velocity impact energies.

As mentioned earlier, the real-time force-displacement data were recorded using a dynamic load cell and a dynamic linear variable differential transformer (LVDT) during the impact experiments, and the representative data have been plotted in Figure 8. In all three plots, “Exp” and “Num” refer to the experimental and numerical results, respectively. All specimens were photographed after the impact events, and the resulting deformations were compared to those numerically simulated. The deformation of the unimpacted side of GLARE and 3DFML specimens are shown in Figure 9 and Figure 10, respectively.

Figure 9 and Figure 10 reveal that the low-impact energy caused mild damage to both material systems, leaving a slight permanent indentation on the back (unimpacted) surfaces. The intermediate-energy level caused a noticeably larger indentation on the back surfaces and caused a small crack on the surface of 3DFML specimens. In contrast, the high-energy level inflicted major damage to both 3DFML and GLARE specimens, while the damage severeness seems to be larger on 3DFML specimens. Large cracks formed on the back surfaces of both material systems, indicating a significant loss of integrity. One can notice that the indentation on the back surface of GLAREs is more noticeable than that on 3DFMLs; this is because GLAREs absorbed the impact energy primarily through flexural deformation, whereas 3DFMLs absorbed energy less through bending but more by causing local through-thickness compressive deformation. The significant flexural deformation of GLAREs is postulated to cause a more significant membrane effect, which in turn aids GLAREs to endure higher impact energy than 3DFMLs.

Under LVI, a larger impact energy induces a more significant deformation to the specimen and, in turn, causes a larger peak impact force. As the impact energy surpasses a critical value that causes the system to lose integrity, the rate of change of the peak impact force slows down, approaching a plateau, which can be observed in Figure 8a. It is noted that the peak forces resulting from intermediate- to high-energy impacts did not vary appreciably in 3DFML in comparison to GLARE. This is because the high-impact energy numerically established for 3DFMLs happened to be slightly higher than the material’s perforation limits, whereas that was not the case for GLARE. For this reason, the 3DFML specimens suffered more severe damage compared to the GLARE specimens.

For the low-impact energy, the smooth responses of GLARE and 3DFML specimens, as shown in Figure 8a, indicate that no significant damage has been caused to the specimens, and the specimens retained their integrity relatively well. The direct contrast can be observed by examination of the specimens’ responses at the high-impact energies (i.e., 30 J for 3DFML and 40 J for GLARE). The sudden decrease in the impact loads indicates that the system has lost its global integrity due to the failure of its constituent(s). The intermediate-impact energy did not cause significant damage to the GLARE specimens; in contrast, the impact force response of the 3DFML specimens turned abruptly into a rough plateau after the steadily increasing rates. Following the same reasoning mentioned above, the intermediate-impact energy caused a small-scale failure of 3DFML’s constituent(s); however, because the residual energy of the impactor was not enough to rapidly propagate the failure, the decline in the impact force is much milder compared to that caused by the large impact energy. As also observed in the figures, the numerical impact failure predictions match the experimentally observed failure of the specimens.

Examination of Figure 8a also reveals that the contact duration becomes shorter as the impact energy is increased. However, the opposite can be observed when the impact energy is increased to a level that compromises the integrity of the specimen because the impactor experiences a reduced impact force, as it causes severe damage to the material and rebounds/stops at a slower pace. Furthermore, by comparing the responses of 3DFML and GLARE in Figure 7a, it can be observed that 3DFML provided a longer impact duration than GLARE, despite the fact that GLARE specimens were subjected to larger impact loads. This phenomenon is caused by the relatively large flexural stiffness but low through-thickness stiffness of the 3DFML. Furthermore, the large thickness of the 3DFML core provided a thicker “crush zone”, which allows it to absorb energy through the prolonged impact duration.

Since the high-impact energy level selected for 3DFML specimens was slightly above the system’s perforation limit, the penetration depth differs from specimen to specimen. However, all specimens managed to stop the impactor successfully. Therefore, the damage and failure modes in the specimens were similar, but relatively different post-penetration responses were observed. The response at this stage in the events is believed to be highly dependent on the impactor shape. As shown in Figure 2b, the impactor used in this study has a slender section with the same diameter as its rounded nose. Once the impactor perforates the target, the resistance force caused by pushing the target decreases to nearly zero. This is because, at this stage, the resistance force is dominated by the friction force between the impactor nose section and the material surrounding the perforated region. As a result, the post-perforation stage becomes a rather lengthy process, making the ever-small differences in the recorded responses more noticeable. In addition, any unintentional variability developed in the material during its fabrication process may result in a slightly different reaction to the applied impact force and the severity of the damage. Nevertheless, since the response after the perforation is not the focus of this study and has limited practical value, this issue will not be scrutinized further in this paper.

#### 5.1.2. Numerical Results

As can be observed through the results presented in Figure 9 and Figure 10, the failure modes were accurately predicted by the numerical approach. As for the real-time impact responses, as also observed in Figure 8, the numerical results also closely agree with the experimental results, especially up to the stage when the system’s integrity has not been affected by the impact. The largest discrepancy between the numerical and experimental results appears in the case of the high-energy impact of 3DFMLs. In this case, the high-impact energy imposes a higher strain rate on the specimens. Since such numerical discrepancies could not be observed in the GLARE specimens, this discrepancy is believed to have been caused due to the foam’s strain-rate effect, which was not accounted for in the numerical models.

The numerical models could also capture the partial perforation that resulted from the high-energy impacts; however, the models underestimated the force response after the onset of the perforations. This may be because the incorporated element size was larger than the crack width, so the numerically predicted crack (mimicked by element erosion) was larger than the real crack width. The underestimated impact force response in turn caused the impactor to maintain its velocity more effectively in the numerical models compared to that observed in the experiments (see Figure 8b).

### 5.2. Post-Impact Inspection

**Figure 11:**
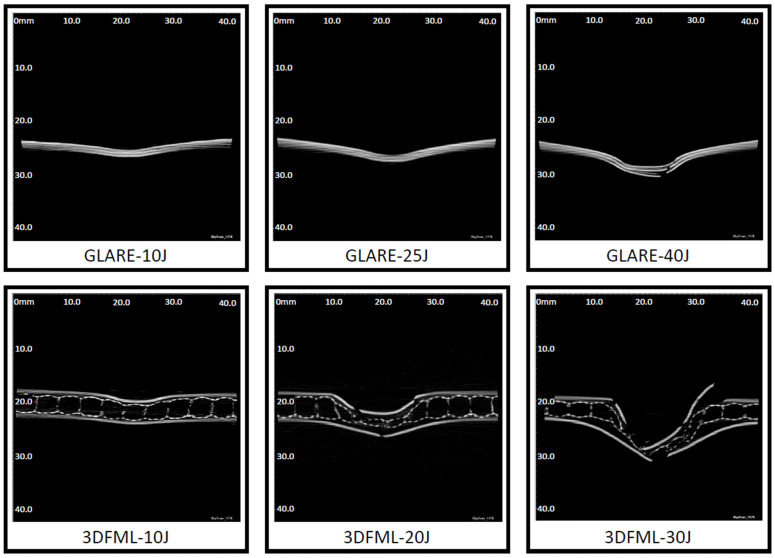
Micro-CT images of impacted specimens.

Micro computerized tomography (micro-CT) scanning was used to generate a series of images of the cross-section along the longitudinal and lateral axis of the specimens. Images that illustrate the cross-sectional deformation of typical specimens under different impact energies are presented in Figure 11. The images include scales (although not quite visible in the reduced dimension images herein), which enable the user to measure the delamination length.

As discussed previously, the impacted GLARE specimens exhibited noticeable flexural deformations compared to their counterparts. The low- and intermediate-impact energies did not cause major material failure, besides the bending deformation in the GLARE specimens; however, interlaminar delamination could be observed through the darker gaps observed between the layers. In contrast, the high-impact energy caused the fracture of all the metallic layers and fiber breakage of all the FRP layers, indicating that the impact energy was near the specimen’s perforation threshold. Moreover, the interlaminar damage was also significantly more extensive.

As for the 3DFML specimens, the low-impact energy caused an indentation on the impacted side and very minor deformation on the unimpacted side. Despite the minor damage on the surface and no obvious sign of delamination, fiber breakage could be observed in the pillars in the vicinity of the impact. Under the intermediate-energy level, both the impacted and unimpacted sides experienced cracking, and the pillars were buckled or broken, rendering the through-thickness collapse of the 3DFRP core. Furthermore, fiber breakage in 3DFRP plies could also be observed. Lastly, under high-impact energy, the specimen suffered severe damage. Significant cracks formed on the metallic layers, and breakages in the plies and collapse of the foam and pillars were substantial. In addition, the specimens showed minor global bending deformation due to the sufficient flexural rigidity of the material system, even under high-impact energy. Moreover, delamination in the lower metal/FRP interface was more prevalent since the impactor seemed to have pressed that layer after perforating through the FML. Lastly, an asymmetric failure response was observed in the 3DFML specimens impacted at the high-energy level. This is postulated to be due to the impactor contacting a region between two rows of supporting pillars, as opposed to directly contacting the spot above a row of pillars.

**Figure 12:**
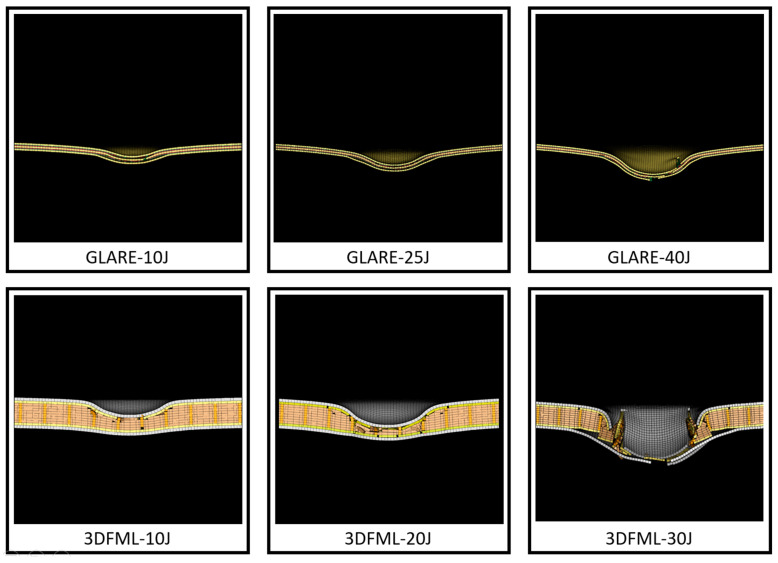
Numerical results that correspond to the locations that were micro-CT scanned.

Figure 12 shows the numerical simulations of the cross-section of the specimens at the same location examined by the micro-CT scan. All numerical models showed excellent agreement with the experimental results. Consistent with the experimental observations, the metallic layers in GLARE stayed intact until they encountered the high-impact energy level (note the more apparent predicted FRP failure and interface delamination). At the high-energy level, the numerical model successfully predicted failures in all three layers of aluminum, and the failure pattern is quite similar to that captured by the CT scan. As for 3DFML specimens, similar to the experimental observations, larger energy left a larger permanent compressive deformation on the 3DFRP core. The numerical simulation also successfully captured the breakage of pillars and plies, as observed experimentally.

**Figure 13:**
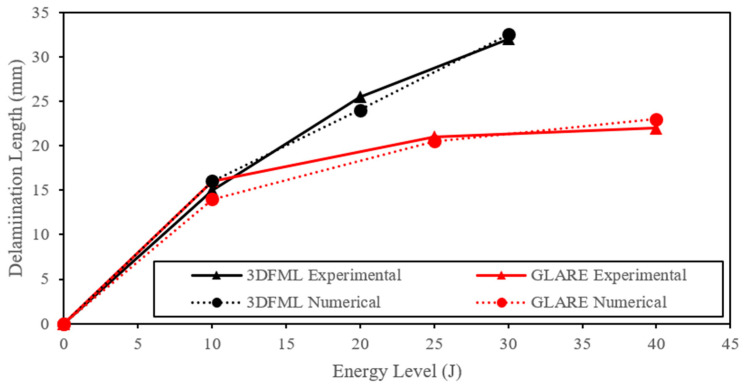
Comparison of the experimentally measured and numerically predicted delamination lengths as a function of applied impact energy.

The delamination length was measured from both the micro-CT scan images and numerical results and was plotted with respect to the energy levels in Figure 13. Both material systems sustained larger delamination as the impact energy increased, but 3DFML sustained larger delaminations. In addition, both materials showed a declining growth rate in delamination length as the impact energy increased. The numerical delamination length was determined using sliced views, as shown in Figure 12, and the “intfor” output files generated by LS-DYNA.

### 5.3. Residual Strength

#### 5.3.1. Modification of the Test Fixture

**Figure 14:**
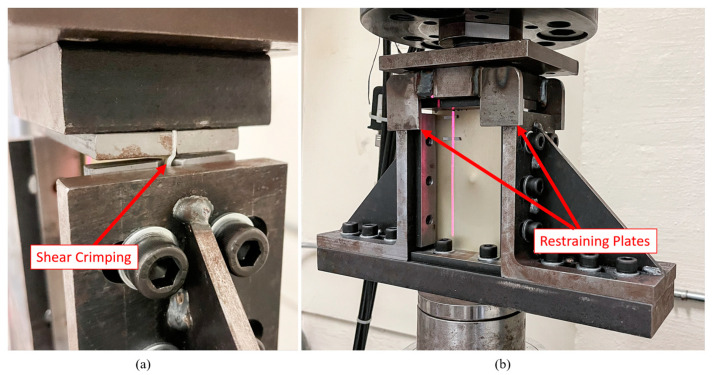
CAI fixture: (**a**) shear crimping of thinner specimens when using the standard. CAI fixture: (**b**) the modified fixture.

The CAI tests were carried out according to ASTM D7137, which recommended a specimen thickness of 5 mm to prevent the buckling of specimens. While the 3DFML specimens’ thicknesses were very close to 5 mm, the GLARE specimens were significantly thinner. The smaller thickness caused a lower buckling resistance and introduced premature shear crimping of the specimens during the compression tests, as illustrated in Figure 14a. The shear crimping occurred at the gap region between the upper grip and the side rails, which left the specimens unsupported during the tests. This issue did not occur at the gap between the lower sliders and side rails because the lower sliders were restrained in the out-of-plane direction with respect to the side rails, as observed in Figure 3. Following this observation, four restraining plates were added to the upper fixture to restrain the specimen in the out-of-plane direction while leaving it free to move in-plane, as shown in Figure 14b. After modifying the standard fixture, the shear crimping issue of the GLARE specimens was overcome. Note that the 3DFML specimens did not experience the above issue due to their sufficient flexural stiffness; only one 3DFML specimen suffered shear crimping during the tests using the original fixture.

#### 5.3.2. CAI Failure Modes

**Figure 15:**
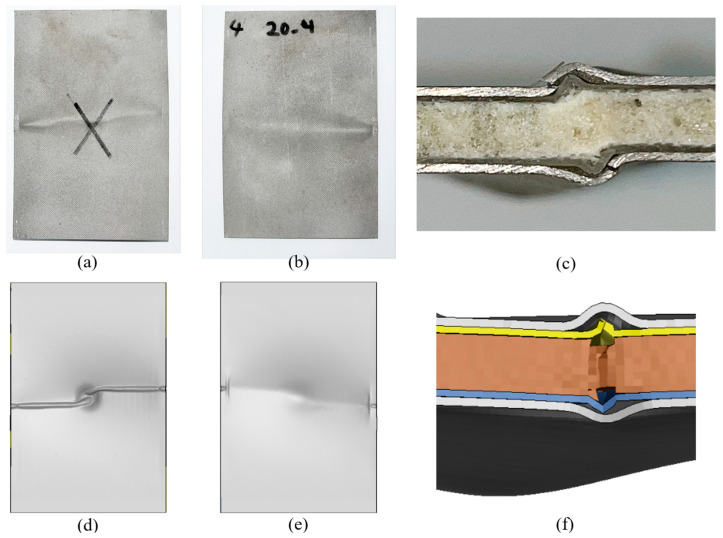
Experimental and numerically predicted CAI failure modes of 3DFML: (**a**) impacted side; (**b**) unimpacted side; (**c**) detailed side view of failure; (**d**) numerical simulation of the impacted side; (**e**) numerical simulation of the unimpacted side; (**f**) side view of failure region simulated numerically.

**Figure 16:**
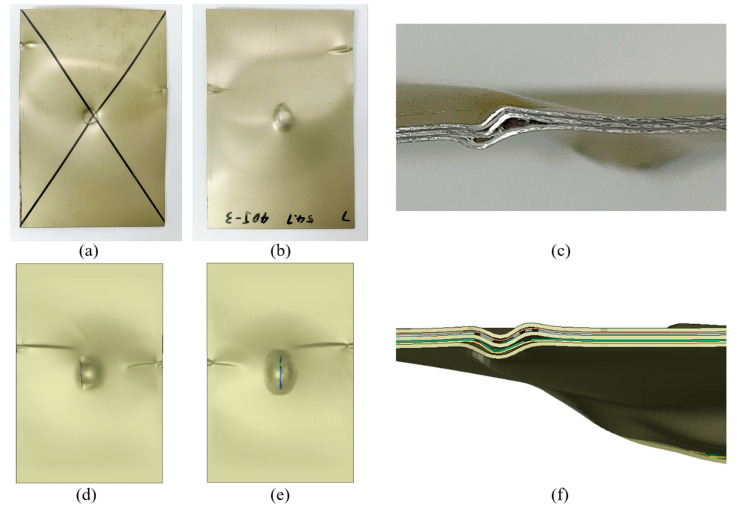
Experimental and numerically predicted CAI failure modes of GLARE: (**a**) impacted side; (**b**) unimpacted side; (**c**) detailed side view of failure; (**d**) numerical simulation of the impacted side; (**e**) numerical simulation of the unimpacted side; (**f**) side view of failure region simulated numerically.

The failure modes were similar in both material systems, regardless of the applied impact energy levels; therefore, the response of a representative specimen per material system is presented in Figure 15 and Figure 16. Each figure also includes the corresponding numerical predictions. According to the “three-letter failure mode codes” of the ASTM D7137 standard, the final failure modes of 3DFML and GLARE are designated as LDM and LGM, respectively. The first, second, and third characters represent failure type, failure area, and failure location, respectively. In this case, L stands for lateral under the “failure type”, D and G stand for at/through damage and damage within the gauge length (away from the damage), respectively, under the “failure area” category, and M stands for middle under the “failure location” category.

The 3DFMLs did not show any noticeable global buckling during the tests, and CAI failure occurred on both sides of the specimens at the impacted region. The failure mode can be assessed as local buckling-induced delamination. Looking closely at the failed regions, one can observe that the 3DFGF plies also buckled, evidencing fiber breakage on both sides of the specimen. The foam was also noticeably locally compressed, and its failure can be clearly observed near the buckled region. Furthermore, the width of the buckling band in the magnesium skin on the impacted side is greater than that on the unimpacted side. Material sections near the side rails were restrained in the out-of-plane direction, thus dodging the buckling stresses and shifting the stresses and causing local failure on the magnesium skin near the side rails. It is, therefore, assumed that this failure mode should not have any bearing on the measured residual strengths. The numerical models predicted similar failure modes. All the local failed phenomena were correctly predicted by the numerical models, indicating the reliability of the numerical modelling framework. This included the buckling of magnesium layers at the impacted region, the higher local buckling amplitude on one side, the slightly tilted buckling shape, the side rails-induced localized failures, and the delamination and fiber breakage of the 3DFGF plies.

The impact tests induced significant local bending deformation (conical shape) at the center of the GLARE specimens. This promoted the global buckling of the GLARE specimens during the CAI tests. Nevertheless, the compressive load caused failure to occur closer to the upper edge, away from the locally impacted and deformed region. It is believed that the failure was affected by the complex deformed shapes of the GLARE specimens. Similar to the failure mode observed in the 3DFML specimens, the CAI-induced failure mode in the GLARE specimens could also be identified as local buckling-induced delamination, with fiber breakage observed in the picture showing the side view of the failed region. The buckled region was smaller than that of 3DFMLs, likely because of the smaller overall thickness of GLARE. Similarly, the side rails also caused concentrated failure to the GLARE skins. The numerical predictions, shown in Figure 16d–f, demonstrate the good accuracy of the numerical model. All the failure characteristics showed excellent agreement with the experimental results, although the locations of the buckled areas were lower than those experimentally observed (closer to the vicinity of impact). This may be due to the selected friction coefficient value in the model being slightly lower than the actual value.

**Figure 17:**
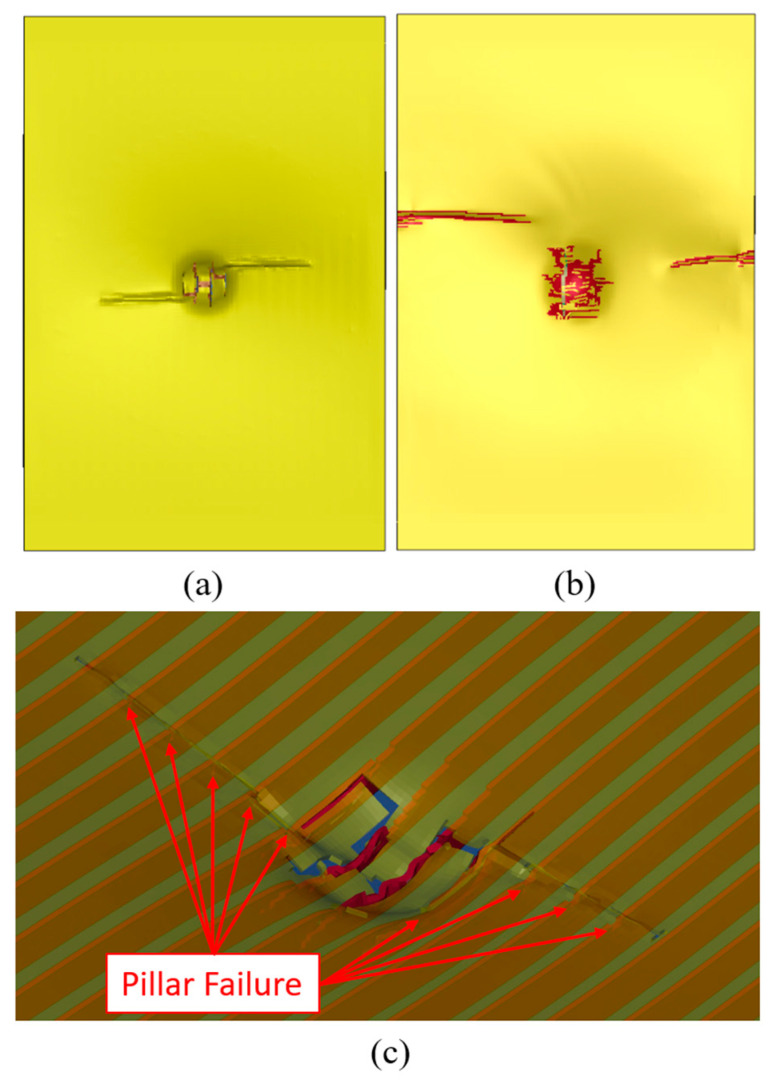
Numerical predictions of the internal failure: (**a**) buckling and cracking on the impacted side of 3DFML skin; (**b**) failure of transverse FRP layer of GLARE on the impacted side; (**c**) failed 3DFGF pillar hidden under buckled skin.

Detailed internal damage in both materials during the compression testing can be observed through the numerical results. Figure 17a shows the buckling failure mode of the 3DFGF biaxial fabric ply (the yellow section) from the impacted side, just after the compression load capacity started decreasing after attaining its maximum. It is evident that not only had the magnesium skins locally buckled, but also the 3DFGF plies buckled along with the magnesium skins. Furthermore, the isoparametric view of the failure region shown in Figure 17c, in which the 3DFGF plies are shown to be semi-transparent, clearly exhibits the failure of the pillars situated directly under the region where the plies buckled. During the compression loading, the two “walls” in the 3DFML tend to buckle, while the pillars resist this tendency (note that a “wall” in this context refers to the combination of the magnesium layer and its underlying 3DFGF ply). When the load increases beyond the limit where the pillars cannot retain the stability of the walls, the pillars break, leading to the buckling of the walls. At that stage, the compression load would decrease as a result of the increased compressive deformation, followed by the delamination of the skins from the pillars. In a subsequent analysis, the influence of pillar strength and bonding strength of 3DFGF to magnesium layers were numerically investigated. The predicted results suggested that the CAI strength was influenced by the strength of pillars, whereas the interface bonding strength had a negligible influence.

One can also notice that the compression failure was initiated from different locations within the specimens. In the case of the 3DFML specimens, the impact caused severe damage to the pillars situated at the location of impact (center of the specimen), leading to the instabilities of the 3DFML walls. Furthermore, the applied compressive load during the CAI test caused additional stress on the already damaged pillars, causing compression failure from the impact damage at the center of the specimen.

In contrast, GLARE’s failure originated from the specimen edges and propagated laterally toward the center. The impact caused a dimple at the center of the GLARE specimen. Once the specimen was subjected to the axial compressive load, the dimpled region deformed further out-of-plane, thereby ununiformly shrinking the damage footprint in the longitudinal direction, causing the failure of the transverse fiber (oriented at 90° to the longitudinal axis) due to the extensive shear stress and the pre-existing compressive stress. Subsequently, the failure progressed toward the dimpled region, and their advancing trajectories turned slightly towards the impact-damaged region and finally joined somewhere near the center of the specimen. Thus, it is believed that the inherent thin configuration of GLARE caused the unexpected failure shift from the center of the specimen (damaged by the initial impact event) to a different location. Furthermore, the described progressive failure theory can also justify the phenomenon of failure shifting from the region damaged by the initial impact toward the upper edges of GLARE specimens.

**Figure 18:**
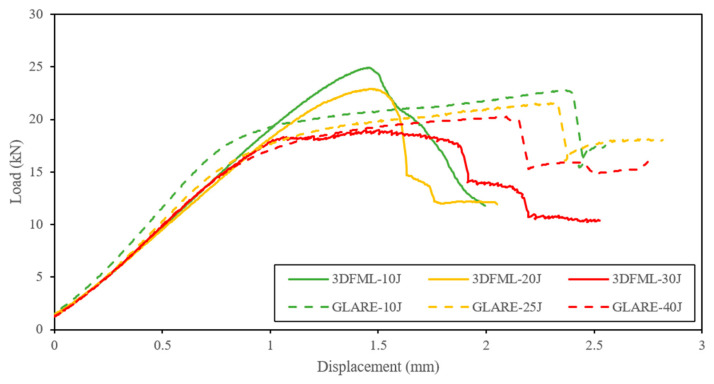
CAI test results.

The experimentally measured load vs. the displacement results of the CAI tests are shown in Figure 18. Both materials showed a more extensive loss of residual strengths due to the increasing impact energy. As previously mentioned, the GLARE configuration was selected to have a similar areal density to our 3DFMLs. Interestingly, the CAI results have revealed that although two FMLs have largely different impact performances, both offered more or less similar residual strength. Furthermore, GLARE specimens exhibited a smaller variation in residual strength under increasing impact energy. In contrast, the residual strength of the 3DFML specimens decreased by a small margin from the low- to intermediate-energy levels, while it decreased even more significantly at the high-energy level.

**Figure 19:**
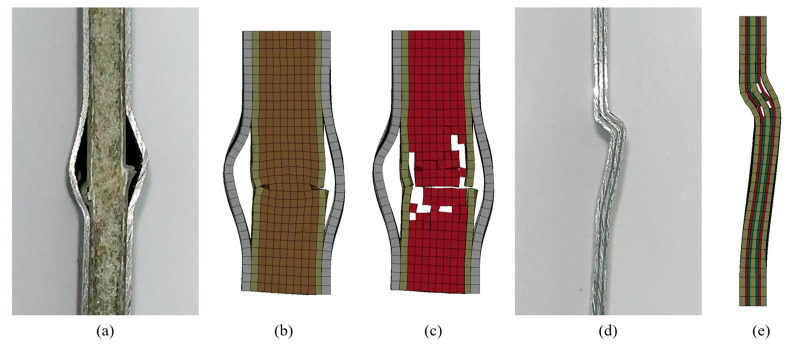
Compression failure mode for virgin specimens: (**a**) actual 3DFML; (**b**) numerically predicted 3DFML; (**c**) numerically predicted pillars’ failure for 3DFML; (**d**) actual GLARE; (**e**) numerically predicted GLARE.

As a comparative investigation, the compressive strengths of the virgin FMLs were evaluated following ASTM D6641. The virgin specimens showed a similar failure mode to the impacted specimens. The failure of the pillars of the 3DFMLs led to the buckling of 3DFGF plies and magnesium layers of 3DFMLs, causing larger scale delamination. Figure 19b shows the numerically predicted compression failure, which matches the experimental observation. Figure 19c shows the side view of the numerically modelled failed specimen, with the foam graphically hidden for clarity. Evidently, the failure of the pillars triggered the buckling of the supporting plies and metallic layers of 3DFMLs. Figure 19d shows the failure mode of the GLARE specimens; as observed, the compressive load caused the failure of the transverse plies and loss of structural integrity, triggering shear crimping failure. The numerical simulation results shown in Figure 19e predicted the same failure mode.

**Figure 20:**
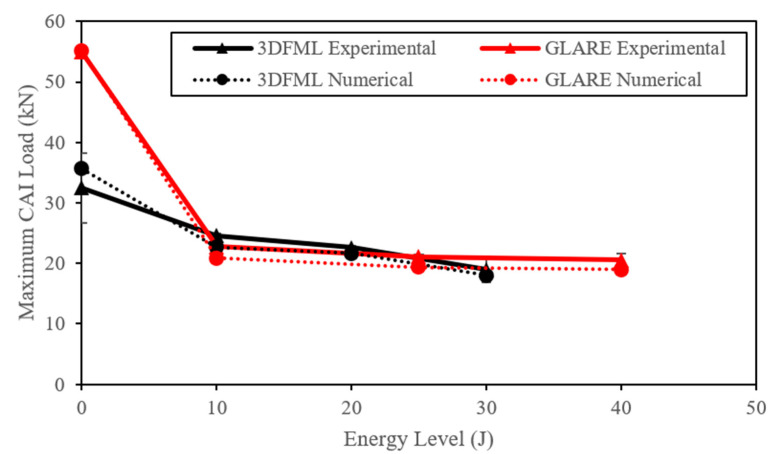
Comparison of the experimental and numerically predicted compressive strengths of virgin and impacted 3DFML and GLARE specimens as a function of impact energy.

The comparison of the compressive residual strength of the impacted specimens and non-impacted specimens is graphically illustrated in Figure 20. Since, as per ASTM D6641, the virgin specimens had a narrower width (12.6 mm and 16 mm for GLARE and 3DFML, respectively) than the specimens that underwent CAI tests (i.e.,100 mm), the maximum load capacity of the virgin specimens was adjusted accordingly and plotted in the graph corresponding to the zero energy impact.

As observed, despite the fact that the virgin GLARE specimens preserved a significantly higher load capacity than their 3DFML counterparts, the impacted GLARE exhibited comparable residual capacity compared to 3DFML. This was expected due to the developed impact-induced dimple on GLARE specimens, which was found to adversely affect their residual load capacity. On the other hand, 3DFML specimens maintained their configuration more effectively. Furthermore, the relatively small thickness of GLARE makes the larger size GLARE specimens significantly prone to buckling during a CAI test, thus substantially lowering its compressive capacity compared to the strength exhibited by the virgin test coupons tested at a much shorter gauge length.

Comparison of the post intermediate- and high-energy impact residual load capacities of both material systems revealed that the residual capacity of the 3DFML significantly decreased, while GLARE maintained its capacity quite well. This is likely due to the more severe damage caused by the high-energy impacts on the 3DFML specimens (the partial perforation compared to the unperforated damage on GLARE specimens). This is because, as stated earlier, the numerically estimated high-impact energy level for 3DFML happened to be slightly greater than the actual perforation threshold of the material system. Overall, however, it can be observed that 3DFML offers better tolerance to impact damage, even though it showed slightly lower impact resistance compared to GLARE.

Furthermore, a relatively larger inconsistency can be observed in the 3DFML experimental results shown in Figure 20. This is believed to be due to flaws unintentionally developed during the fabrication process (e.g., voids and non-uniform resin wetting/distribution), and also whether the impact point was directly above a wall of pillars or between two walls.

Additionally, the numerical models’ results were fairly accurate in predicting the CAI residual capacity and failure modes of both material systems. The predicted capacity for all cases is within 10% of the experimentally measured values. Interestingly, the trends in the curves illustrated in Figure 13 and Figure 20 are similar but in reverse.

The Residual Reduction Index (RRI), defined by Equation (9), can be used to compare the CAI performance of coupon-sized specimens, regardless of the potentially different undamaged compressive strength.

(9) $$RRI=1-\frac{F_{r}}{F_{u}}$$

**Figure 21:**
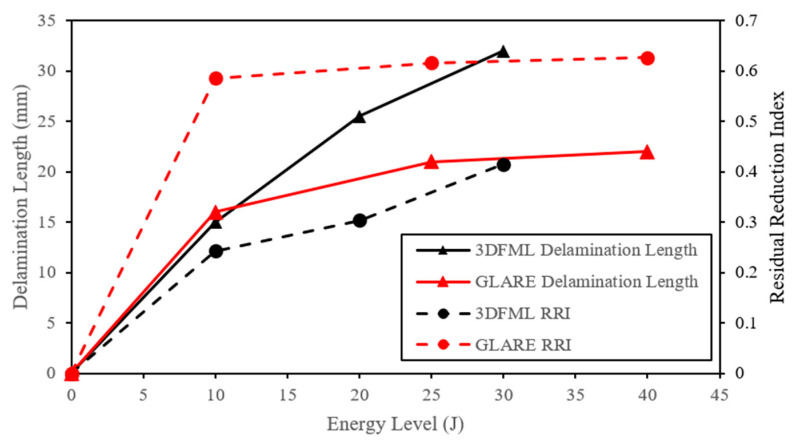
A comparison between delamination length and RRI.

In the above equation, $F_{u}$ is the undamaged compressive load capacity evaluated as per ASTM D7137, and $F_{r}$ is the residual load capacity measured according to ASTM D7137. In other words, the RRI index refracts the degree of the degradation of a material’s compressive strength (or integrity) due to pre-inflicted impact damage. The variations in RRI for the two material systems as a function of the impact energy are shown in Figure 21, along with the corresponding post-impact delamination length. As observed, GLARE shows a higher RRI for all impact energies, indicating that compared to 3DFML, GLARE showed a comparatively lower tolerance to impact loadings.

Furthermore, there is a distinct correlation between delamination length and RRI for both materials, particularly for GLARE. It is plausible that the extensive flexural deformation induced the delamination in the vicinity of the impact; therefore, one may conclude that the delamination length could represent the severity of the impact-induced damage. By examining the CAI failure modes, one can also observe the correlation between the failure and the severity of the associated impact-induced deformation. Therefore, the closer correlation between delamination length and RRI for GLARE would be understandable. In contrast, a weaker correlation between delamination length and RRI can be observed for 3DFMLs, since the CAI failure in 3DFML was dominated by pillar strength and was mostly independent of the bonding strength of 3DFML.

A simple and practical parameter is suggested here for predicting an FML panel’s residual load capacity using the ASTM D7137 measured results based on the following assumptions. It is assumed that the impact fixture used in the experiment has an adequate diameter so that the generated impact damage would be at the same scale as that developed by the same size impactor striking a large panel in service. It is also assumed that the test panel does not experience global buckling; accordingly, the reduction in the residual compressive load capacity should be on the same scale in both cases. Therefore, the following equation can be used to calculate the reduction in the residual strength.

(10) $$F_{rr\left(E,d\right)}=F_{u}-F_{r\left(E,d\right)}$$

where $F_{rr}$ represents the residual strength after the material has been subjected to an impact of energy E with an impactor of diameter d. The magnitudes of the reduction in residual load capacity have been reported in Table 4.

## 6. Conclusions

This work investigated the CAI responses of 2D and 3D fiber metal laminates (i.e., GLARE3/2-0.3 and 3DFML) with similar areal densities through a comprehensive coupled experimental and numerical study. The outcomes of this study and suggestions for further research are summarized as follows.

- The CAI performance of GLARE and 3DFML was successfully evaluated using ASTM D7137 guidelines; however, minor modifications had to be applied to the standard test fixture.
- Micro-CT scans were found to be an effective and worthy non-destructive inspection technique for observing internal damage in such complex hybrid material systems.
- The impact energy levels ranged from low to high, with the selection of high-impact energy close to each material system’s perforation threshold, which was established by numerical simulations.
- The impacted GLARE and 3DFML specimens lost approximately 60% and 40% of their original compressive load-carrying capacity, respectively. GLARE’s significant loss of load capacity is attributed to its relatively small thickness.
- The developed numerical models predicted the impact and post-impact responses of both material systems accurately and effectively. In addition, the predicted failure modes closely matched the experimental results, confirming the integrity of the numerical models.
- The GLARE specimens showed better impact resistance and post-impact compressive load capacity compared to 3DFML; however, due to their large impact-induced deformations, their CAI residual capacity was closely comparable with 3DFMLs. Given the fact that 3DFML possesses comparatively significantly higher flexural properties, 3DFML is deemed to be an effective alternative to GLARE in various engineering applications.
- The developed numerical models were successfully used to establish the influences of the critical and limiting parameters that govern the post-impact compressive load capacity of 3DFML. The developed modelling framework can be effectively used to evaluate the response of different 3DFML configurations and optimize their impact resistance and CAI performance in the future.
- Two equations were proposed for calculating the Reduction in Residual Capacity and Residual Reduction Index. These simple equations can be used to relate the post-impact residual load capacity of real-world FML panels to the CAI performance of laboratory-size specimens. However, the universality of these equations should be further investigated.

## Figures and Tables

**Table 1:** MAT_24 parameters.

	$\mathrm{Density}\;\left(kg/m^{3}\right)$	Elastic Modulus $\left(Pa\right)$	Poisson’s Ratio	Yield Strength $\left(Pa\right)$	FAIL
Mg AZ31B-H24	1772	$4.48\times 10^{10}$	0.35	$1.36\times 10^{8}$	0.1411
Al 2024-T3	2796	$7.31\times 10^{10}$	0.33	$3.26\times 10^{8}$	0.2641

**Table 2:** Values of the parameters used in MAT_54.

Properties *	3DFGF Biaxial Fabric	3DFGF Pillars	UD S-Glass Prepreg
ρ $\left(kg/m^{3}\right)$	1750	1750	1746
$E_{11}\left(Pa\right)$	$9.00\times 10^{9}$	$3.00\times 10^{9}$	$5.24\times 10^{10}$
$E_{22}\left(Pa\right)$	$9.00\times 10^{9}$	$1.00\times 10^{9}$	$1.60\times 10^{10}$
$E_{33}\left(Pa\right)$	$2.55\times 10^{9}$	$1.00\times 10^{9}$	$1.60\times 10^{10}$
$\nu_{21}$	0.05	0.05	0.0944
$\nu_{31}$	0.05	0.05	0.0944
$\nu_{32}$	0.05	0.05	0.42
$G_{12}\left(Pa\right)$	$1.00\times 10^{9}$	$1.00\times 10^{9}$	$4.57\times 10^{9}$
$G_{23}\left(Pa\right)$	$1.00\times 10^{9}$	$1.00\times 10^{9}$	$3.03\times 10^{9}$
$G_{31}\left(Pa\right)$	$1.00\times 10^{9}$	$1.00\times 10^{9}$	$4.57\times 10^{9}$
$\varepsilon_{22}^{ult}$	0.08	0.12	0.0990
$\gamma_{12}^{ult}$	0.12	0.12	0.1328
$\varepsilon_{11t}^{ult}$	0.08	0.108	0.2175
$\varepsilon_{11c}^{ult}$	−0.08	−0.108	−0.1290
$\sigma_{11c}\left(Pa\right)$	$1.73\times 10^{8}$	$8.00\times 10^{7}$	$1.688\times 10^{9}$
$\sigma_{11t}\left(Pa\right)$	$1.73\times 10^{8}$	$8.00\times 10^{7}$	$2.812\times 10^{9}$
$\sigma_{22c}\left(Pa\right)$	$1.73\times 10^{8}$	$8.00\times 10^{7}$	$3.938\times 10^{8}$
$\sigma_{22t}\left(Pa\right)$	$1.73\times 10^{8}$	$8.00\times 10^{7}$	$1.286\times 10^{8}$
$\tau_{11c}\left(Pa\right)$	$3.00\times 10^{7}$	$3.00\times 10^{7}$	$1.519\times 10^{8}$

* Designations as per LS-DYNA User’s Manual [27].

**Table 3:** CZM tiebreak contact parameters.

	NFLS (Pa)	SFLS (Pa)	PARAM	$\mathrm{ERATEN}\;(J/m^{2})$	$\mathrm{ERATES}\;(J/m^{2})$	CT2CN	CN (Pa)
3DFML	$5.90\times 10^{7}$	$2.30\times 10^{7}$	1	1500	2000	0.4286	$3.5\times 10^{12}$
GLARE	$1.33\times 10^{8}$	$5.18\times 10^{7}$	1	3375	4500	0.4286	$7.9\times 10^{12}$

**Table 4:** Reduction in residual compressive load capacity for GLARE and 3DFML corresponding to the tested energy levels.

	10 J	20 J	25 J	30 J	40 J
GLARE-3/2	32.20 kN	-	33.87 kN	-	34.42 kN
3DFML	7.893 kN	9.861 kN	-	13.48 kN	-

## Data Availability

Data available on request.
